# Supplementary material for: Simultaneous representation of a spectrum of dynamically changing value estimates during decision making
Source: Nat Commun. 2017 Dec 5;8:1942. doi: 10.1038/s41467-017-02169-w (PMC5717172; doi:10.1038/s41467-017-02169-w)
Supplement: Supplementary file 1 — Supplementary Information [file 41467_2017_2169_MOESM1_ESM.pdf]

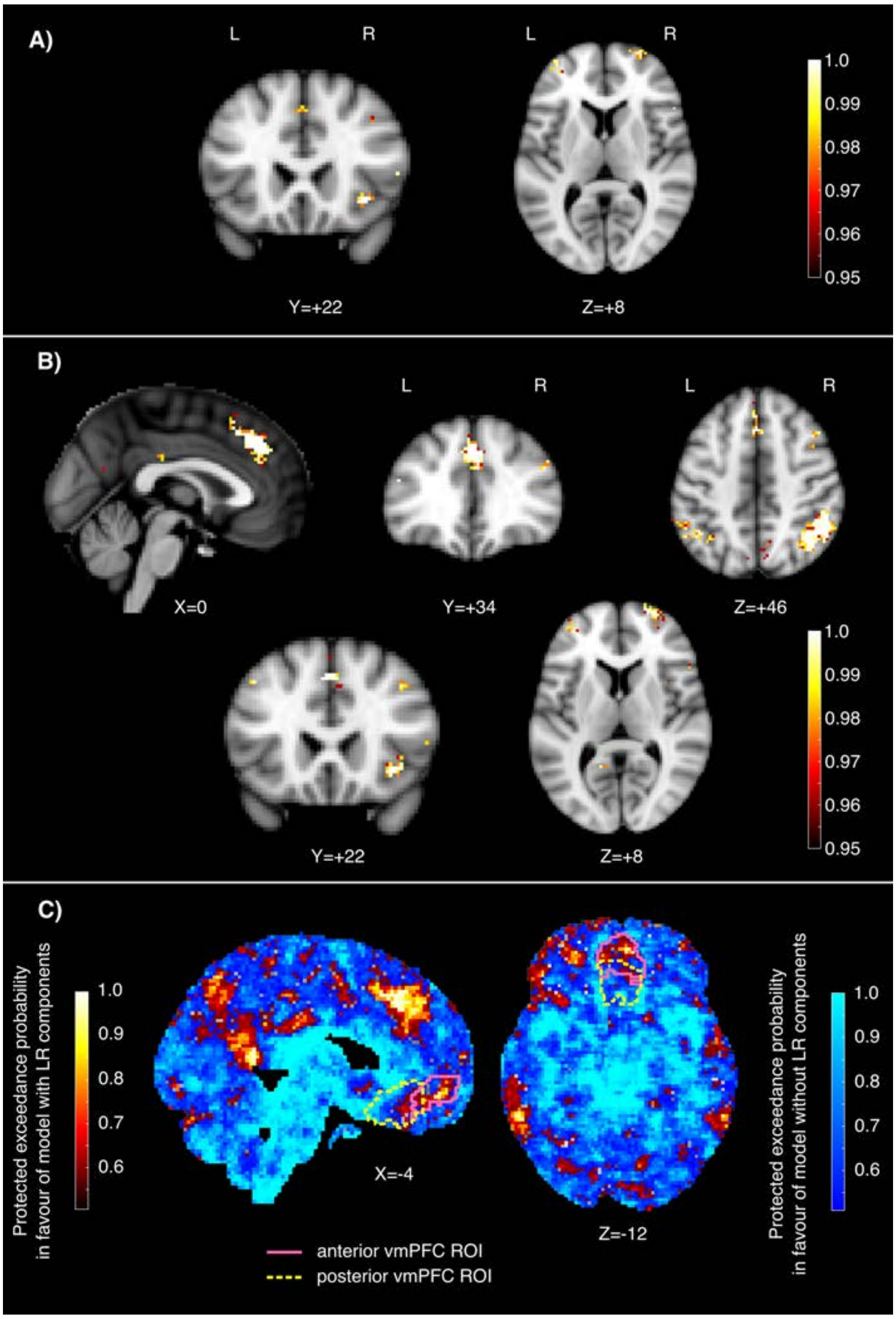

Supplementary Fig. 1. Regions showing high evidence for coding multiple LRs. We used random effects Bayesian model comparison to compare how a GLM with only our standard regressors compared to a GLM additionally containing the first three principal components of a singular value decomposition. The figure shows voxels with high evidence for coding multiple LRs (posterior exceedance probability > 0.95). A) The rFO and bilateral FPl regions showed high evidence of coding reward probability estimates based on multiple LRs. B) Using a GLM containing a win/loss outcome regressor, the effects in dACC, IPL, rFO and FPl are emphasized even more (see Supplementary Note 1). C) The model comparison for the GLMs shown in A), here with the entire range of exceedance probabilities in favour of the model without principal components (blue-lightblue) and with principal components (red-yellow). LRs also have an impact on a number of voxels in vmPFC, but with lower evidence. The pink and yellow outlines show the ant. and posterior vmPFC ROIs.

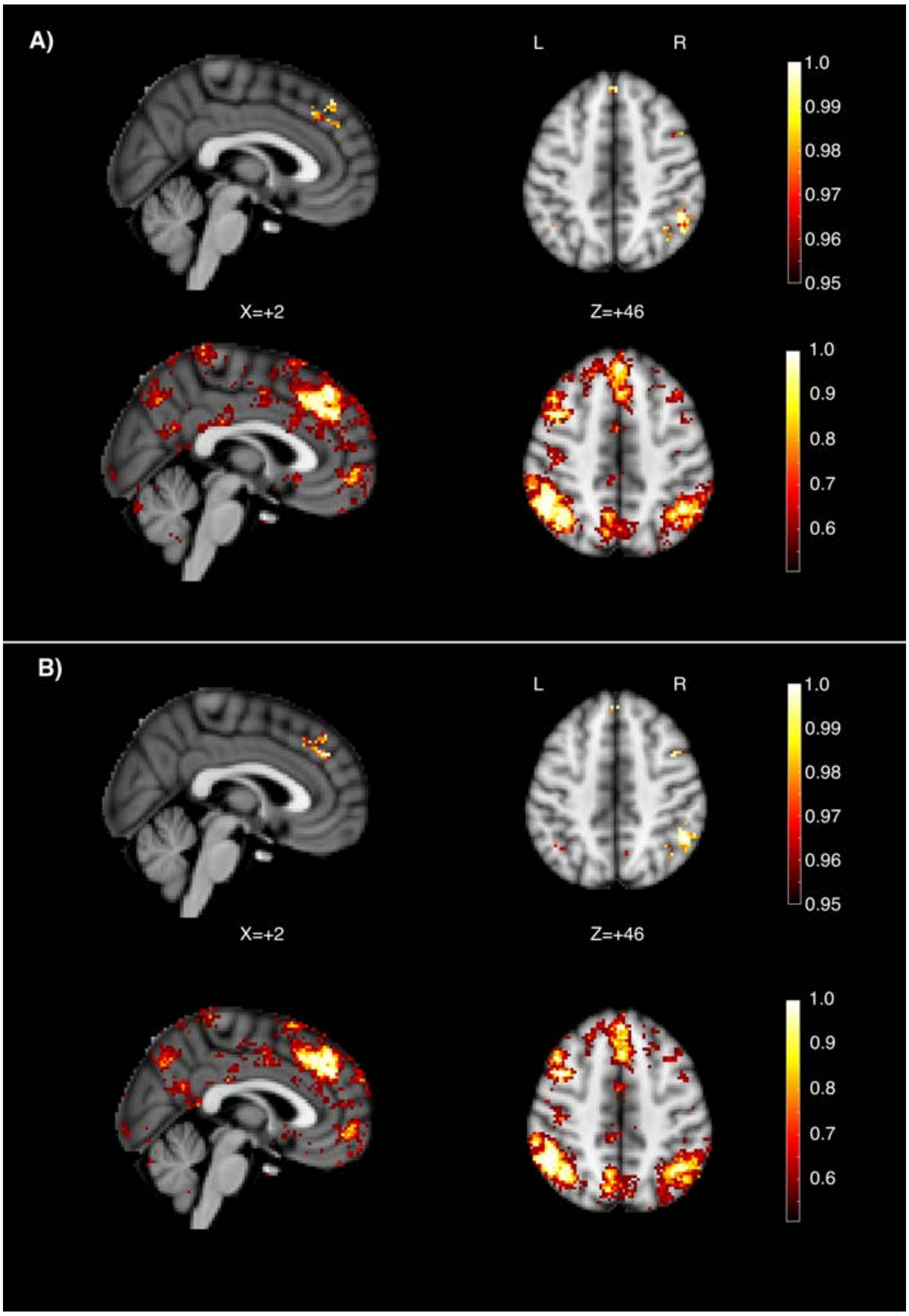

Supplementary Fig. 2. Regions showing high evidence for coding multiple LRs. We used random effects Bayesian model comparison to compare how a GLM first three principal components of a singular value decomposition compared against two other models (see Supplementary Note 1). A) The figure shows voxels with high evidence for coding multiple LRs (top row: posterior exceedance probability > 0.95, bottom row: posterior exceedance probability > 0.50) compared against a model with choice values generated from the behaviorally optimal LR, i.e. the LR that would lead to gain-maximizing behavior. B) The figure shows voxels with high evidence for coding multiple LRs (top row: posterior exceedance probability > 0.95, bottom row: posterior exceedance probability > 0.50) compared against a model with choice values generated from the LR fitted to subjects' behavior.

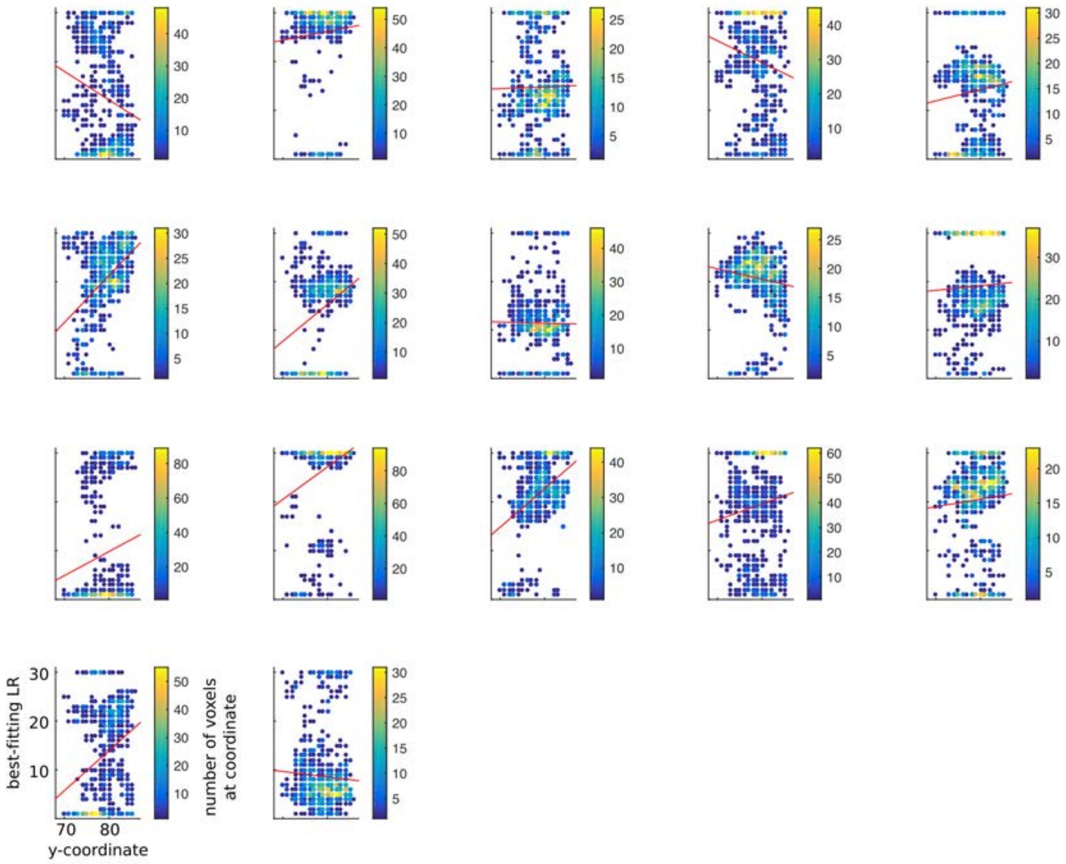

Supplementary Fig. 3. Topography of LR distributions in the individual subjects' ACC. Each voxel's best-fitting LR is plotted against its position on the y-coordinate. Note that the color of the dots reflects the number of voxels having a given combination of values (see color bars next to graph). Red lines: Regression of all voxels' best-fitting LR against their y-coordinate. Despite the interindividual variability, there is a significant rostro-caudal gradient at the group level when testing all regression coefficients against zero.

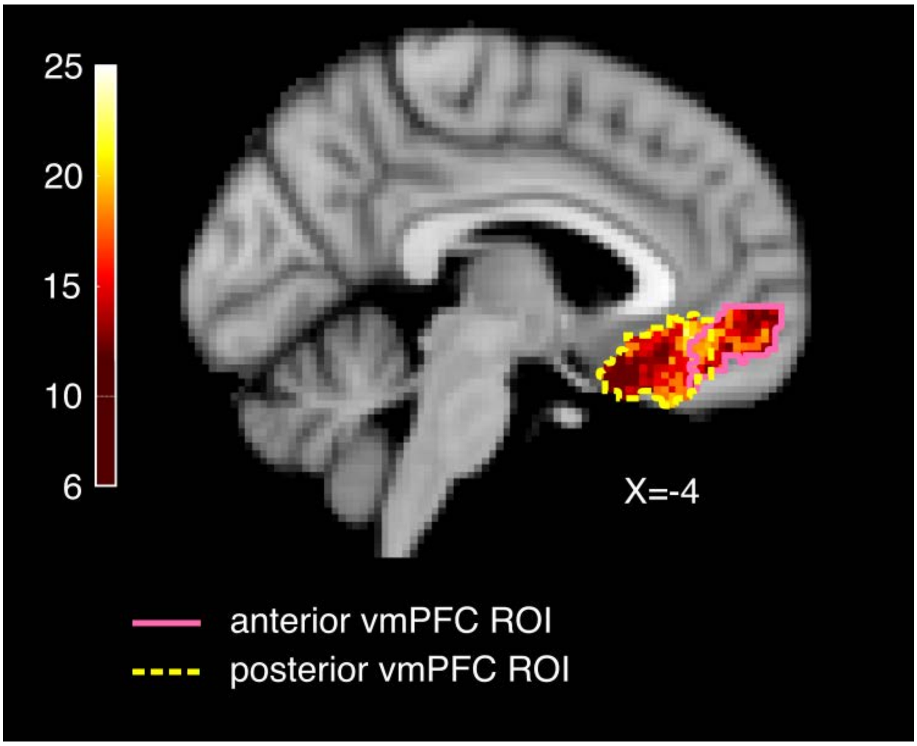

Supplementary Fig. 4. Topographic maps of LRs in anterior and posterior vmPFC. Diverse estimates of the reward probability exist in the vmPFC regions as well (see Supplementary Note 3). In comparison to Fig. 4 in the main manuscript, a larger number of low LR voxels are apparent (for better comparability of the color scale between Fig. S4 and Fig. 4, the color scale is the same between LRs 10 to 25, here all values below 10 are kept in a constant dark red).

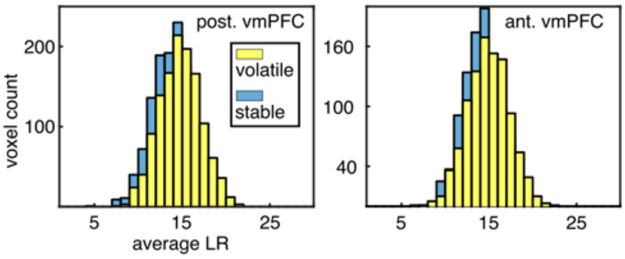

Supplementary Fig. 5. Dynamic changes in LR between stable and volatile sub-session. Distribution of number of voxels with best-fitting LRs. There is no significant difference between the mean of the two distributions in either region (see Supplementary Note 3).

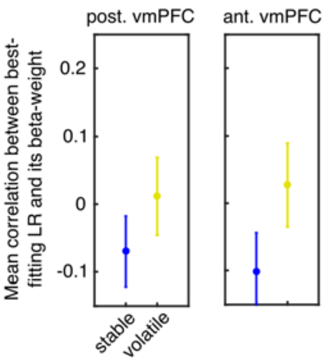

Supplementary Fig. 6. Change in the correlation between beta-weights of the best-fitting LR regressors and the best-fitting LR between sub-sessions. There is no significant difference in the correlation between the two sub-sessions in either region **(**see Supplementary Note 3). Error bars: Standard error of the mean.

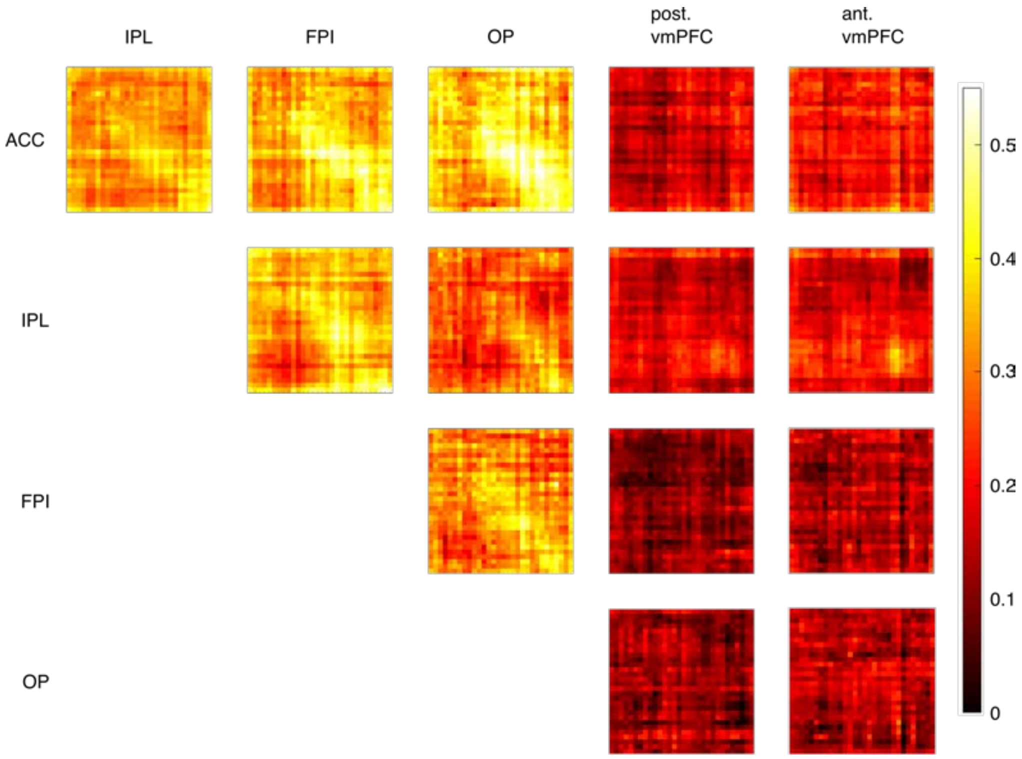

Supplementary Fig. 7. LRs as an organizing principle in the information exchange between regions. Correlation plots depicting the correlation, between regions, of the residual BOLD time course averaged over all voxels with the same best-fitting LR, averaged over all subjects (see Supplementary Note 4). Results of tests examining the significance of the relationship (t-tests of z-transformed correlation coefficients of the correlation between subjects' z-transformed time-course correlation coefficients with their closeness to the diagonal [negative Euclidian distance]) are reported in Supplementary Table 1. We did not test for correlation between anterior and posterior vmPFC since the ROI's overlapped with 576 voxels.

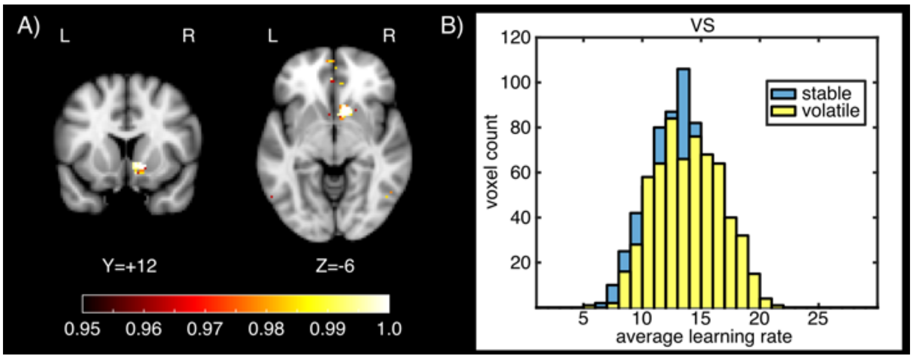

Supplementary Fig. 8. Ventral striatum showing high evidence for coding multiple LRs during the outcome phase. A) The figure shows voxels with high evidence for coding multiple LRs (posterior exceedance probability > 0.95) during the outcome phase in right VS. B) The distribution of best-fitting LRs was stable between the stable and volatile sub-sessions. T-test over each subject's mean change in best-fitting LRs between volatile and stable sub-session: t_{16}=-0.631, p=0.537

Supplementary Table 1. Testing correlation of residual time-courses with closeness to diagonal

		IPL	rOP	bil. FPl	ant. vmPFC	post. vmPFC
ACC	t_{16}	-2.944	-4.239	-3.340	2.438	2.836
	p	0.005	0.003	0.002	0.987	0.994
IPL	t_{16}		-3.134	-3.125	-0.942	-0.922
	p		0.003	0.003	0.180	0.185
rOP	t_{16}			-3.850	-0.112	-0.475
	p			0.001	0.456	0.321
FPl	t_{16}				1.312	2.282
	p				0.896	0.982

Supplementary Table 1. Testing whether the correlation, between regions, of residual time-courses of voxels is higher for more similar LRs. In every subject, the mean residual time courses of voxels with the same LR in every region (residual of a GLM explaining the BOLD time course with our standard GLM and all LR regressors) were correlated with the mean residual time courses of all voxels with the same LRs in all other regions. These correlation coefficients were z-transformed and then correlated with their closeness to the diagonal (negative Euclidian distance) in the correlation matrix. These were again z-transformed and all subjects' z-values of all pairs of regions were tested against zero with one-tailed t-tests. We did not test for correlation between anterior and posterior vmPFC since the ROI's overlapped with 576 voxels.

Supplementary Table 2. Testing for differences between the mean correlation of residual time-courses

	ACC x FPI 0.364	ACC x OP 0.407	IPL x FPI 0.346	IPL x OP 0.287	FPI x OP 0.327	ACC x ant. vmPFC 0.227	ACC x post. vmPFC 0.179	IPL x ant. vmPFC 0.198	IPL x post. vmPFC 0.181	FPI x ant. vmPFC 0.145	FPI x post. vmPFC 0.109	OP x ant. vmPFC 0.154	OP x post. vmPFC 0.129
ACC x IPL 0.332	t=-1.205 p=1.000	t=-2.669 p=1.000	t=-0.584 p=1.000	t=1.691 p=1.000	t=0.204 p=1.000	t=3.702 p=0.079	t=5.440 p=0.000	t=5.317 p=0.001	t=5.883 p=0.000	t=6.668 p=0.000	t=8.605 p=0.000	t=6.500 p=0.000	t=7.853 p=0.000
ACC x FPI 0.364		t=-1.574 p=1.000	t=0.738 p=1.000	t=2.945 p=0.607	t=1.556 p=1.000	t=4.915 p=0.002	t=6.706 p=0.000	t=6.753 p=0.000	t=7.301 p=0.000	t=7.969 p=0.000	t=10.078 p=0.000	t=7.828 p=0.000	t=9.313 p=0.000
ACC x OP 0.407			t=2.369 p=1.000	t=4.306 p=0.014	t=3.130 p=0.376	t=6.095 p=0.000	t=7.790 p=0.000	t=7.898 p=0.000	t=8.397 p=0.000	t=8.981 p=0.000	t=10.976 p=0.000	t=8.857 p=0.000	t=10.266 p=0.000
IPL x FPI 0.346				t=2.462 p=1.000	t=0.898 p=1.000	t=4.591 p=0.006	t=6.512 p=0.000	t=6.610 p=0.000	t=7.198 p=0.000	t=7.871 p=0.000	t=10.216 p=0.000	t=7.729 p=0.000	t=9.387 p=0.000
IPL x OP 0.287					t=-1.675 p=1.000	t=2.131 p=1.000	t=3.862 p=0.050	t=3.552 p=0.120	t=4.158 p=0.022	t=5.089 p=0.001	t=6.917 p=0.000	t=4.888 p=0.003	t=6.153 p=0.000
FPI x OP 0.327						t=3.871 p=0.049	t=5.791 p=0.000	t=5.787 p=0.000	t=6.397 p=0.000	t=7.150 p=0.000	t=9.440 p=0.000	t=6.991 p=0.000	t=8.603 p=0.000
ACC x ant. vmPFC 0.227							t=1.607 p=1.000	t=1.054 p=1.000	t=1.667 p=1.000	t=2.750 p=0.996	t=4.261 p=0.016	t=2.512 p=1.000	t=3.540 p=0.124
ACC x post. vmPFC 0.179								t=-0.732 p=1.000	t=-0.080 p=1.000	t=1.147 p=1.000	t=2.563 p=1.000	t=0.877 p=1.000	t=1.826 p=1.000
IPL x ant. vmPFC 0.198									t=0.724 p=1.000	t=2.011 p=1.000	t=3.703 p=0.079	t=1.732 p=1.000	t=2.876 p=0.726
IPL x post. vmPFC 0.181										t=1.330 p=1.000	t=2.921 p=0.647	t=1.041 p=1.000	t=2.108 p=1.000
FPI x ant. vmPFC 0.145											t=1.339 p=1.000	t=-0.293 p=1.000	t=0.594 p=1.000
FPI x post. vmPFC 0.109												t=-1.685 p=1.000	t=-0.816 p=1.000
OP x ant. vmPFC 0.154													t=0.925 p=1.000

Supplementary Table 2. Testing for differences in the average correlation values of residual time-courses. In every subject, the mean residual time courses of voxels with the same LR in every region (residual of a GLM explaining the BOLD time course with our standard GLM and all LR regressors) were correlated with the mean residual time courses of all voxels with the same LRs in all other regions. These correlation coefficients were z-transformed. The average z-value for every correlation matrix for every subject was tested against the average z-values from the correlation matrix between another pair of regions. Numbers below each pair of regions in first column and first row are the mean over every subject's average z-value for that pair. Significantly different average correlations are highlighted in grey. For all but three comparisons, the average correlation between pairs of regions with high

(table legend continued from previous page) evidence for LR-coding (ACC, IPL, FPI, OP) are significantly higher than the average correlation between pairs where one of the regions is anterior or posterior vmPFC (red box). We did not test for correlation between anterior and posterior vmPFC since the ROI's overlapped with 576 voxels. P-values are Bonferroni corrected.

In order to ascertain that this effect was not driven by a correlation between beta-weights and the best-fitting LR of that regressor, we tested whether the best-fitting LR was correlated with the beta-value of that regressor across the entire session. However, in none of the regions were the beta-values of the best-fitting LR regressors correlated with the best-fitting LR (t-test of each subject's z-transformed correlation coefficient against 0. dACC: mean z-score = 0.09, $t_{16} = 1.516$, $p = 0.149$; IPL: mean z-score = 0.013, $t_{16} = 0.203$, $p = 0.841$, posterior vmPFC: mean z-score = 0.031, $t_{16} = 0.643$, $p = 0.529$; anterior vmPFC: mean z-score = 0.011, $t_{16} = 0.241$, $p = 0.812$).

Supplementary Note 1: Evidence for LR-information in different brain regions

Besides the dACC and IPL, which were our *a-priori* defined regions of interest, the right operculum (rFO) and bilateral lateral frontopolar (FPl) regions also showed high evidence of coding LRs (Supplementary Fig. 1A). The main focus of our analysis was on the distribution of LRs during the decision phase, however, we also wanted to investigate whether we would find comparable distributions with the estimated prediction error (probability prediction error, not weighted with reward magnitude) during the outcome phase (Supplementary Fig. 7). Using the prediction error regressors during the outcome phase, we could not use a regressor modeling the actual outcome (win or loss), given its high correlation with the prediction error regressors. However, when using a different GLM where we used the same probability estimate regressor during the outcome phase as we used for the decision phase, we were able to include a win/loss outcome regressor. Using this model, the random effects Bayesian Model comparison showed even stronger evidence for the coding of LRs in two of our ROIs (dACC and IPL) and in rFO and FPl (Supplementary Fig. 1B). Thus, while this model is not optimal for investigating LR regressor distributions based on prediction errors during the outcome phase, it does suggest that adding a win/loss outcome regressor removes some noise from the signal during the decision phase, resulting in even stronger effects of the model comparison in favor of the model containing LR information in those regions.

We ran two further analyses to corroborate the results. First, we compared our singular value (multiple LR) model against a model using optimal behavioural LRs (Supplementary Fig. 2A). The optimal LR we defined as the LR that would lead to optimal (gain-maximizing) choice behavior. This was then used to generate choice values and added as a regressor to the GLM. Second, we compared it against a model using the LR that was fit to subject behaviour (Supplementary Fig. 2B). For the fitting we used a hierarchical Bayesian model fitting procedure (Stan (mc-stan.org) with Matlab interface). Since the group level average parameter fit has been shown to give the best power for group level fMRI[4], we used this learning rate of 0.25 to as an LR regressor to compare against the model with the three singular value regressors. Both approaches yield similar (albeit slightly weaker) results.

Supplementary Note 2: Defining the Regions of Interest

For dACC, the ROI included bilateral areas 24a/b, d32 as well as the bilateral anterior rostral zones of the cingulate motor areas[5]. For IPL, the ROI included inferior parietal lobule areas c and d as defined by Mars and colleagues[6]. The atlas only contains IPL regions for the right hemisphere, we therefore mirrored the regions along the midline to create masks for the left hemisphere. Since the anatomical masks are defined by white matter connectivity, they do not cover the entire width of the cortex in any area. Therefore, the dACC and vmPFC masks were extended with 2 voxels medially, while the IPL masks were extended laterally and caudally to ensure that all grey matter voxels were covered by the masks.

In order to test whether the effects we show in the dACC and IPL are ubiquitous mechanisms in all brain regions, we performed the same tests in another region associated with value signals, the vmPFC. Because most studies find value signals in more anterior regions of vmPFC and because we found a strong effect of the reward magnitude of the chosen option in posterior vmPFC, we decided to create two separate ROIs, anterior and posterior vmPFC. The ROIs were created in the same manner as the dACC and IPL ROIs by the overlap of anatomical masks from the connectivity-based parcellation atlases (posterior vmPFC: area 14, anterior vmPFC: area 11m, **http://www.rbmars.dds.nl/CBPatlases.htm**)[5] and significant activity changes associated with the reward magnitude of the chosen option during decision (Supplementary Fig. 1C).

Supplementary Note 3: Examining LR representation and dynamic adaptation to changes in the environment in vmPFC.

It was possible to fit value estimates with various LRs to voxels in anterior and posterior vmPFC (Supplementary Fig. 4) but none of the beta-coefficients of any regressor relating LR to a spatial dimension were significantly different from 0 in either region.

We performed the same tests on the posterior and anterior vmPFC regions that we had applied to dACC and IPL in order to ascertain whether the effects we had found were ubiquitous across all areas of the brain. First, we found that the distribution of best-fitting LRs did not shift between the two sub-sessions in either vmPFC region (Supplementary Fig. 5, compare to Fig. 5). Average LR difference (stable minus volatile) in posterior vmPFC was -0.96 (t-test between each subject's mean change in LR's $t_{16} = 0.50$, p=0.624) and -0.701 in anterior vmPFC ($t_{16} = -0.3538$, p=0.7281).

Second, we found no shift in the correlation between the best-fitting LR and the beta-weight of that regressor as we had in the dACC (Supplementary Fig. 6, compare to Fig. 6A). In the posterior vmPFC the mean difference between the correlation in stable and volatile sub-session was -0.081 ($t_{16} = -0.948$, p = 0.357, z-scores stable sub-session against zero: $t_{16} = -1.344$, p = 0.198, z-scores volatile sub-session against zero: $t_{16} = 0.200$, p = 0.844) while in the anterior vmPFC it was -0.128 ($t_{16} = -1.310$, p = 0.209, z-scores stable sub-session against zero: $t_{16} = -1.775$, p = 0.095, z-scores volatile sub-session against zero: $t_{16} = 0.432$, p = 0.671).

Supplementary Note 4: LRs as an Organizational Principle of Interregional Interaction between all regions with high evidence for LR information and both vmPFC ROIs

We had shown that voxels that code recent reward probability experienced with one time constant in one brain region (dACC) interact preferentially with voxels with similar time constants in the other ROI (IPL). This effect (intuitively visible as high values [yellow-white colors] along the diagonal of the correlation plots) also existed in the interaction amongst the other regions with high evidence for LR coding (FPI and rOP), but not in the interaction with our vmPFC ROIs (Supplementary Fig. 7, Supplementary Table 1). Furthermore, with only few exceptions, average correlations (mean over average of every subject’s correlation matrix) were higher for correlations between two regions with high evidence for LR information coding (e.g. dACC and FPI) compared to correlations between one of these regions and one of the vmPFC ROIs (e.g. dACC and posterior vmPFC) (Supplementary Table 2).

Supplementary Note 5: Dynamic Adaptation of the Distribution of LRs to Changes in the Environment

While the focus of the current investigation was the dynamic representation of value estimates during the decision, we also tested whether we could find evidence that the distribution of best-fitting LRs for the generation of prediction errors during the outcome phase adapted to changes in the environment. While the ventral striatum (VS) showed high evidence for coding prediction errors derived from reinforcement learning (Supplementary Fig. 8A, analysis akin to the one described in the main manuscript, only with principal components from an SVD over LR regressors generating prediction errors), it did not show any shift in the distribution depending on the sub-session (Supplementary Fig. 8B).

Supplementary Methods 1: Preprocessing

Functional images were first spatially smoothed (Gaussian kernel with 6mm full-width half-maximum) and temporally high-pass filtered (3 dB cut-off of 100s). We used the Brain Extraction Tool (BET) from FSL[1] on the high-resolution structural MRI images to separate brain matter from non-brain matter. The resulting images guided registration of functional images to the high-resolution structural images using boundary-based registrations (6 degrees of freedom) and subsequently using affine linear (12 degrees of freedom) and non-linear registration between the structural image and a high-resolution structural template in Montreal Neurological Institute (MNI)-space[2,3].

Supplementary References

1. Smith, S. M. Fast robust automated brain extraction. *Hum. Brain Mapp.* **17,** 143–155 (2002).
2. Greve, D. N. & Fischl, B. Accurate and robust brain image alignment using boundary-based registration. *NeuroImage* **48,** 63–72 (2009).
3. Jenkinson, M., Bannister, P., Brady, M. & Smith, S. Improved Optimization for the Robust and Accurate Linear Registration and Motion Correction of Brain Images. *NeuroImage* **17,** 825–841 (2002).
4. Ahn, W.-Y., Krawitz, A., Kim, W., Busmeyer, J. R. & Brown, J. W. A Model-Based fMRI Analysis with Hierarchical Bayesian Parameter Estimation. *J. Neurosci. Psychol. Econ.* **4,** 95–110 (2011).
5. Neubert, F.-X., Mars, R. B., Sallet, J. & Rushworth, M. F. S. Connectivity reveals relationship of brain areas for reward-guided learning and decision making in human and monkey frontal cortex. *Proc. Natl. Acad. Sci.* **112,** E2695–E2704 (2015).
6. Mars, R. B. *et al.* Diffusion-Weighted Imaging Tractography-Based Parcellation of the Human Parietal Cortex and Comparison with Human and Macaque Resting-State Functional Connectivity. *J. Neurosci.* **31,** 4087–4100 (2011).
